# Photo-Mediated Ultrasound Therapy (PUT) for the Treatment of Deep Cutaneous Vasculature

**DOI:** 10.1109/ojuffc.2025.3604391

**Published:** 2025-09-02

**Authors:** MINGYANG WANG, JEFFREY S. ORRINGER, YANNIS M. PAULUS, XINMAI YANG, XUEDING WANG

**Affiliations:** 1 University of Michigan, Ann Arbor, MI 48109 USA; 2 Johns Hopkins University, Baltimore, MD 21218 USA; 3 The University of Kansas, Lawrence, KS 66045 USA

**Keywords:** Deep cutaneous vasculature, port-wine stains, photomechanical effect, photoacoustic, photo-mediated ultrasound therapy

## Abstract

Traditionally, pulsed dye laser (PDL) therapy has been used to treat cutaneous blood vessels in patients with port-wine stain (PWS) birthmarks. PDL therapy, however, has limited treatment depth, and frequently results in suboptimal therapeutic outcomes when used to treat deep cutaneous blood vessels. We have developed photo-mediated ultrasound therapy (PUT), a hybrid cavitation-based anti-vascular technology combining nanosecond light pulses with ultrasound bursts and demonstrated its great potential in treating deep cutaneous vessels. This study explored the feasibility of PUT as an alternative to traditional PDL therapy for deep cutaneous vascular treatment in a clinically relevant chicken wattle model. PUT was employed to induce cavitation in blood vessels by using different light fluence and ultrasound pressure combinations. Theoretical modeling and *in vitro* experiments were first conducted to validate and optimize parameters for PUT treatment targeting deep vasculature. PUT treatments were then performed in a chicken wattle model using an experimental setup, and outcomes were assessed by using polarized dermoscope, optical coherence tomography angiography (OCT-A) imaging, and histopathological analyses. The results demonstrated that PUT can effectively penetrate the entire thickness of chicken wattle tissue, which is about 3 mm, and significantly reduce blood vessel density by 45.20% with a light fluence 10–100 times less than the fluence used in traditional PDL therapy. OCT-A imaging showed that local blood perfusion was significantly reduced, and the reduced blood perfusion persisted for at least 7 days post-treatment in the treated areas. Histopathological analyses based on H&E, CD31, and Russell-Movat Pentachrome (RMP) stains confirmed effective and selective vascular damage through the entire thickness of chicken wattle without causing collateral thermal damage. In conclusion, PUT can effectively eliminate blood vessels with a treatment depth up to 3 mm whereas the 3 mm treatment depth demonstrated in this study was only limited by the chicken wattle model. By leveraging the deep tissue penetration of ultrasound and the flexibility in treatment parameter selection, PUT can effectively treat deep cutaneous vasculature using reduced light fluence and thereby minimize collateral damage in skin tissues. Thus, PUT holds great potential for treatment of cutaneous vascular anomalies such as PWS.

## I. INTRODUCTION

Ultrasound induced mechanical stress has been one of the common mechanisms utilized in ultrasound therapy to achieve desired biological effects for many years. Multiple mechanical stress-based ultrasound therapeutic methods have been developed with a broad range of applications including kidney stone treatment, lens removal in cataract surgery, ultrasound targeted drug delivery, and focused ultrasound tissue ablation [1], [2]. As a secondary mechanism following ultrasound exposure, acoustic cavitation plays a critical role in the mechanical stress-based ultrasound therapeutic modalities. Acoustic cavitation typically starts from gas nuclei in liquid media. Stable cavitation can be induced by utilizing low ultrasound power with the systemic injection of microbubbles (e.g. blood-brain barrier opening using focused ultrasound (FUS) [3]). Alternatively, pre-existing bubbles can serve as cavitation nuclei when high-intensity ultrasound is applied to induce inertial cavitation directly (e.g. lithotripsy [4] and histotripsy [5]).

In recent years, we developed a hybrid cavitation-based therapy by using synchronized nanosecond light pulses and ultrasound bursts, termed photo-mediated ultrasound therapy (PUT). While light alone can induce cavitation in liquid media through either optical breakdown or heating effects [6], [7] (e.g., intraocular photodisruption [8]), PUT is based on the photomechanical effect of light by using nanosecond light pulses to generate photoacoustic waves in selected optical absorbers (e.g., blood vessels) within the confinements of thermal relaxation and stress relaxation. By precisely controlling the spatiotemporal synchronization between the nanosecond light pulse induced photoacoustic waves and the applied ultrasound bursts, cavitation can be induced with lower light fluence and lower ultrasound pressure when compared to pure light based or pure ultrasound based cavitation methods [9]. Based on the unique treatment mechanism, anti-vascular PUT holds potential for many medical applications, including those in cosmetic and procedural dermatology [10].

According to the International Society for the Study of Vascular Anomalies, vascular anomalies are classified into tumors, simple malformations, and combined malformations [11]. Capillary vascular malformation or port-wine stains (PWS) are congenital low-flow vascular malformations of the skin and are the most common cutaneous vascular malformation condition [12]. Pulsed dye laser (PDL) treatment is the current clinical standard and the first-line therapeutic method for treating PWS. In PDL treatment, 595 nm laser is widely used as it matches an absorption peak of hemoglobin, while 0.45–3 ms laser pulse duration is typically used to match the thermal relaxation time of blood vessels [13]. The carefully selected laser wavelength and pulse width allow for good selective vascular obliteration. However, a substantial fraction of the PWS patient population suffers from suboptimal therapeutic outcomes [14]. Clinical studies have pointed out that suboptimal treatment from PDL was mainly due to its limited penetration depth, which is about 0.6 mm when using typical 595 nm lasers whereas PWS lesions can extend to a depth of up to 3 mm beneath skin surface [15], [16]. To improve the treatment depth, multiple studies have been carried out with longer wavelength lasers. Alexandrite lasers at 755 nm [17] and Nd-YAG lasers at 1064 nm [18] have been used in clinical trials. However, due to the lower optical absorption of oxygenated hemoglobin at these longer wavelengths, laser therapy with these devices requires much higher fluences to destroy malformation vessels, causing increased safety concerns about collateral thermal damage in nearby tissues.

As a potential alternative therapy for PWS, PUT utilizes cavitation generated by the synergistic application of nanosecond light pulses and ultrasound bursts. This allows for at least an order of magnitude reduction in the required laser fluence compared to PDL therapy and thereby offers a potentially much improved safety. Our previous studies have demonstrated the anti-vascular efficacy of PUT using the rabbit ear model [19], [20] and chicken wattle model [21]. The present study aims to further explore the potential of PUT in the treatment of deep cutaneous vasculature. As demonstrated in this study, by leveraging the superior tissue penetration capability of ultrasound, PUT treatment of deep PWS lesions can be achieved by focusing the ultrasound energy at greater depths to compensate for the diminished light fluence that can be effectively delivered to deep tissue.

## II. MECHANISM

The underlying mechanism for PUT is cavitation induced by synchronized light pulses and ultrasound bursts. Previous theoretical studies on cavitation bubble dynamics and cavitation-induced stresses during PUT have identified three distinct phases during the interaction of light pulses and ultrasound bursts within blood vessels [22], [23]. The three distinct phases could be outlined as: 1) in the initial phase, a pulsed light induced photoacoustic wave, synchronized with the peak negative pressure (PNP) of a ultrasound wave, triggers bubble nucleation; 2) in the subsequent phase, the formed bubble expands through rectified diffusion process in the ultrasound field; and 3) in the third phase, the bubble either breaks up or reaches a stable equilibrium radius and undergoes non-inertial oscillation. In the present study, we used an established theoretical model [24] to investigate how light fluence and ultrasound PNP influence pre-existing bubbles during PUT by implementing the light and ultrasound parameters utilized in our current experiments. The mathematical model and parameters applied are detailed in Supplementary Information 1.

**FIGURE 1.**
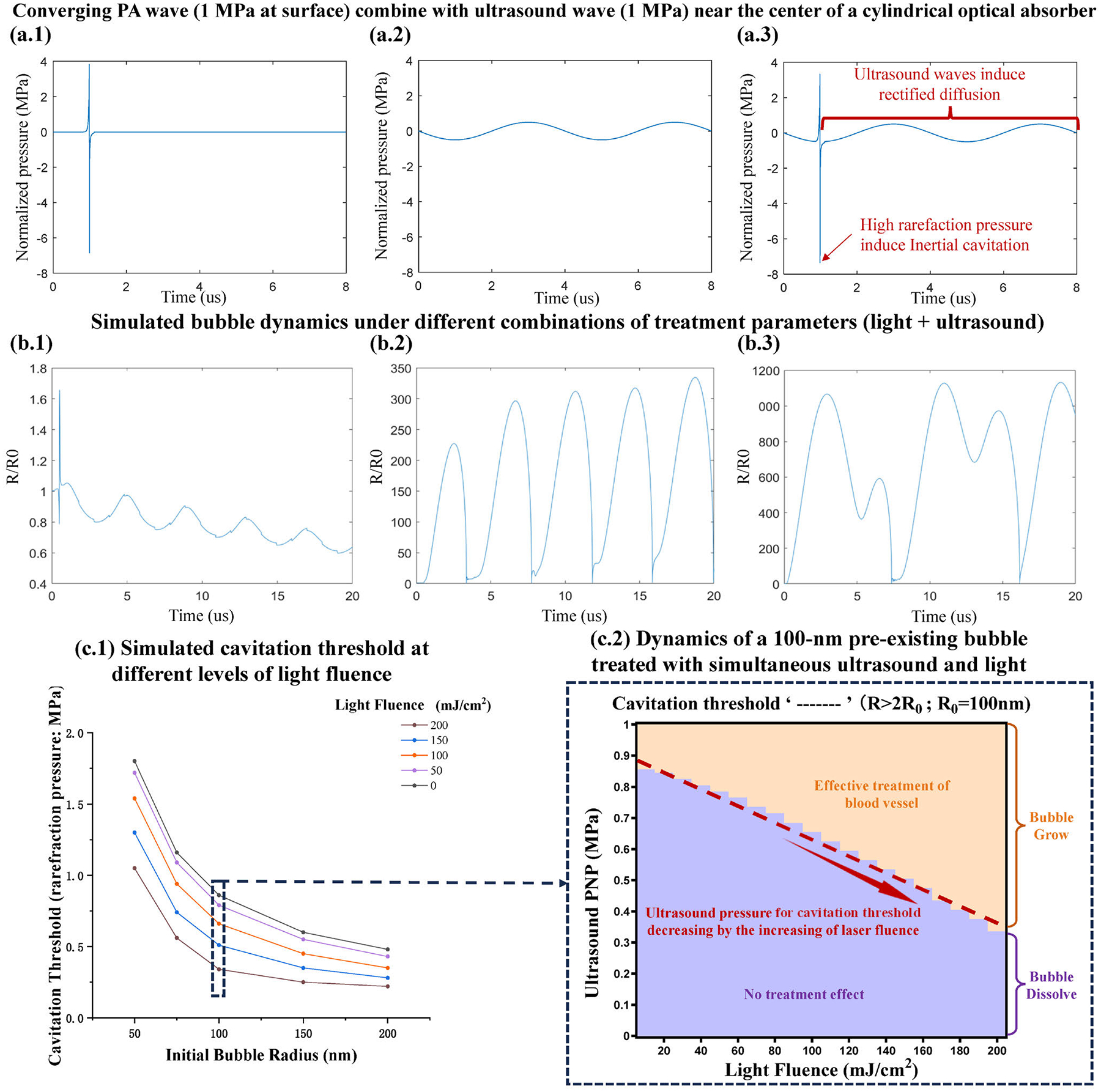
Theoretical modeling of the pre-existing bubble dynamics under different light fluence and ultrasound peak negative pressure (PNP) during PUT treatment of a blood vessel. (a.1) Simulated photoacoustic (PA) wave near the center of a blood vessel with a diameter of 0.1 mm when illuminated by a 3-ns light pulse at 1064 nm wavelength. (a.2) Concurrently applied ultrasound burst with 0.25 MHz frequency and 1 MPa pressure amplitude. (a.3) Combined PA waveform in (a.1) and ultrasound wave in (a.2), where the PA wave is synchronized at the negative phase of an ultrasound cycle. (b.1-b.3) Simulated bubble size evolution under different combinations of treatment parameters, including 0.6 MPa ultrasound and 100 mJ/cm^2^ light fluence (b.1), 0.8 MPa ultrasound and 120 mJ/cm^2^ light fluence (b.2), and 1.0 MPa ultrasound and 120 mJ/cm2 light fluence (b.3). R/R0 stands for dynamic bubble size R over its initial size R0. (c.1) Simulated rectified diffusion threshold (i.e., cavitation threshold) for different initial bubble sizes and at different levels of light fluence. (c.2) Simulated dynamics of a 100 − nm pre-existing bubble under the treatment of different combinations of ultrasound rarefaction pressure and light fluence, where the bubble dissolves (i.e., no treatment effect) in the blue region and grows (i.e., effective treatment of blood vessel) in the yellow region. The dashed red line indicates the ultrasound pressure for cavitation threshold which decreases with the increased light fluence.

Fig. 1.a.1 shows the simulated photoacoustic waveform observed near the center of a blood vessel with a diameter of 0.1 mm when illuminated by a 3-ns light pulse at 1064 nm wavelength. Fig. 1.a.2 shows the concurrently applied ultrasound burst with 0.25 MHz frequency and 1 MPa peak to peak pressure amplitude. The light pulse and the ultrasound burst are synchronized with a precisely controlled delay so that the light-induced photoacoustic pulse is superimposed onto the negative phase of an ultrasound cycle, as shown in Fig. 1.a.3. Depending on the light and ultrasound parameters, the combined acoustic pressure may or may not reach the rectified diffusion cavitation threshold for a 100 nm radius pre-existing bubble. For example, an ultrasound pressure of 0.6 MPa combined with a light fluence of 100 mJ/cm^2^ does not reach the rectified diffusion threshold, resulting in the dissolution of the pre-existing bubble as shown in Fig. 1.b.1. In contrast, an ultrasound pressure of 0.8 MPa combined with a light fluence of 120 mJ/cm^2^ surpasses the rectified diffusion threshold, resulting in an explosive growth of the bubble as shown in Fig. 1.b.2. Furthermore, an ultrasound pressure of 1.0 MPa combined with a light fluence of 120 mJ/cm^2^ causes the bubble to expand sufficiently to induce period doubling as shown in Fig. 1.b.3 [25].

Considering that the pre-existing bubble size may vary, Fig. 1.c.1 illustrates the rectified diffusion threshold (i.e., cavitation threshold) in terms of ultrasound pressure for different levels of light fluence and different initial bubble sizes. The result shows that the increased light fluence can reduce the ultrasound pressure needed for cavitation, which agrees with the findings in our previous studies [24]. Assuming a pre-existing bubble with a radius of 100 nm, we simulated the dynamics of the bubble under the treatment with different combinations of ultrasound rarefaction pressure (0–1 MPa) and light fluence (0–200 mJ/cm^2^), as shown in Fig. 1.c.2. The result shows a region (highlighted with orange color) where the pre-existing bubble grows under PUT treatment. In this region, the bubble, continuously driven by the ultrasound burst, can cause effective and selective treatment of the blood vessel. Fig. 1.c.2 also shows a region (highlighted with purple color) where the pre-existing bubble dissolves under PUT treatment. In the region, there will be no treatment effect to the blood vessel. A dashed red line between these two regions indicates the rectified diffusion threshold in terms of the ultrasound pressure as a function the light fluence. Once again, we can see that the increased light fluence can reduce the ultrasound pressure needed for cavitation, and vice versa. This result also suggests that, at the skin surface where the light fluence is strong, less ultrasound pressure is needed to produce cavitation; while in deep skin where the light fluence is weaker due to the strong optical attenuation in overlying tissues, the likelihood of cavitation can be increased by applying relatively higher ultrasound pressure. Therefore, using the optimal combination of light fluence and ultrasound pressure, cavitation determining the treatment effect of PUT can be induced in both superficial and deep skin at the same time.

## III. METHOD

### A. PHANTOM STUDY

**FIGURE 2.**
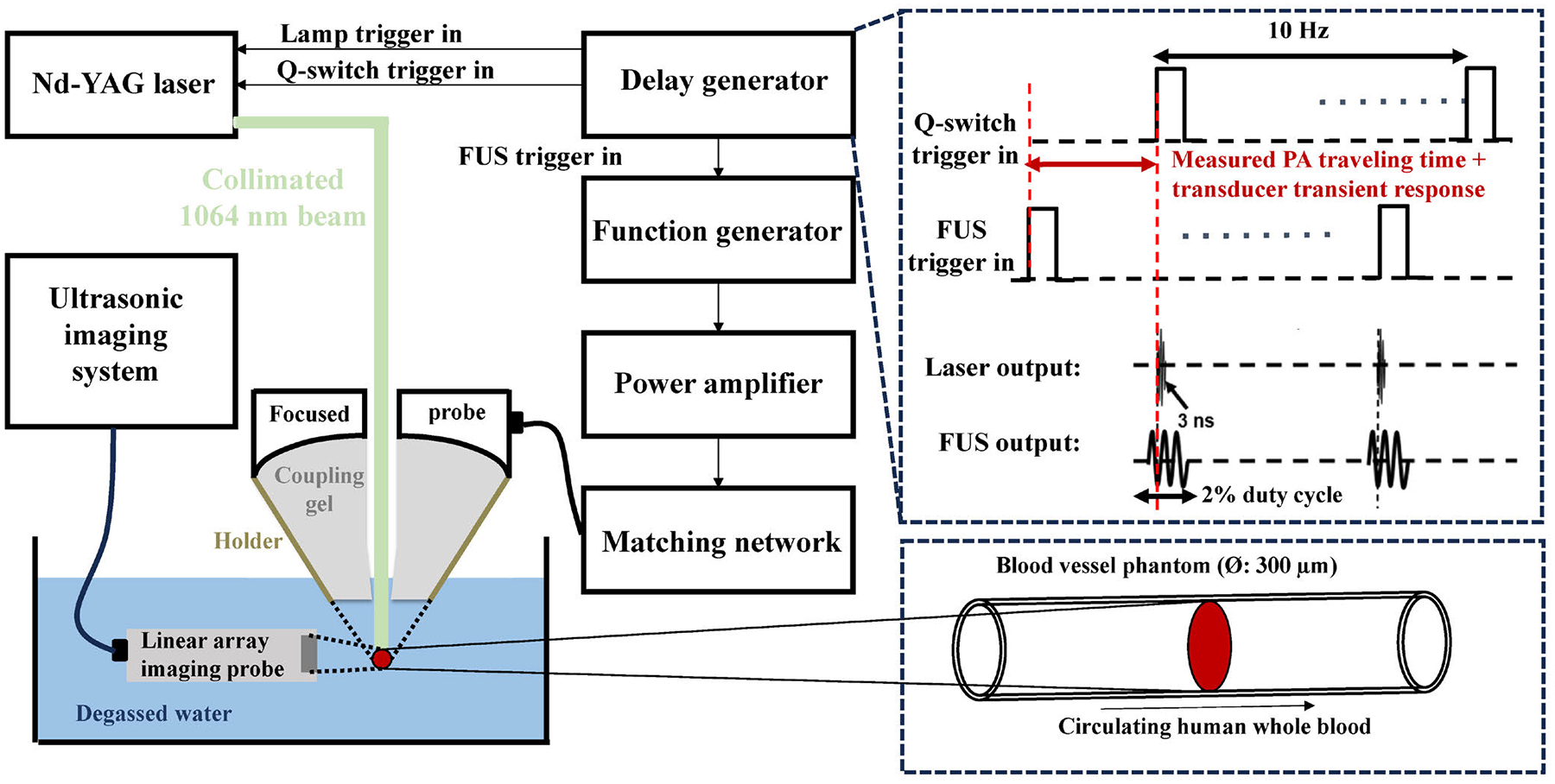
Phantom validation of cavitation activity in a blood vessel phantom treated by PUT under active cavitation detection (ACD).

The schematic of the phantom experiment setup is illustrated in Fig. 2. The nanosecond light pulses with a 3 nm pulse width and pulse repetition rate of 10 Hz were from an Nd:YAG laser (Continuum PowerliteDLS8010, Santa Clara, CA) working at 1064 nm wavelength. The light beam was directed to the center (aperture size of 22.60 mm) of a focused ultrasound transducer with a focal length of 39.49 mm, a −6 dB focal width of 6.04 mm, and working at a center frequency of 0.25 MHz (H-117, Sonic Concepts, Bothell, WA). In this study, the transducer generated 2 millisecond long ultrasound bursts with a 2% duty cycle. The transducer was powered by a radio-frequency power amplifier (2100L, ENI, Rochester, NY) through an impedance matching network provided by Sonic Concepts. A pulse delay generator (Model DG355, Stanford Research Systems) was employed to synchronize the triggers for the laser and the ultrasound systems, ensuring that the light pulse reached the target during one of the negative phases of the ultrasound bursts [9].

The target was a blood vessel mimicking phantom which was a soft and optically transparent silicone tube (inner diameter: 0.3 mm, outer diameter: 0.6 mm, Liveo^™^Silicone Laboratory Tubing, Fisher Scientific) filled with human whole blood obtained from the University of Michigan Blood Center. Driven by a pump, the blood was circulated through the tube at a speed of 1 cm per second. During the measurement, the tube was immersed in a water bath filled with degassed water. For real-time active cavitation detection during PUT, the blood vessel was imaged continuously using an ultrasound imaging system (ZS3, Zonare Medical Systems, Inc., Mountain View, CA) in the B-mode working with a 10 MHz linear probe.

**FIGURE 3.**
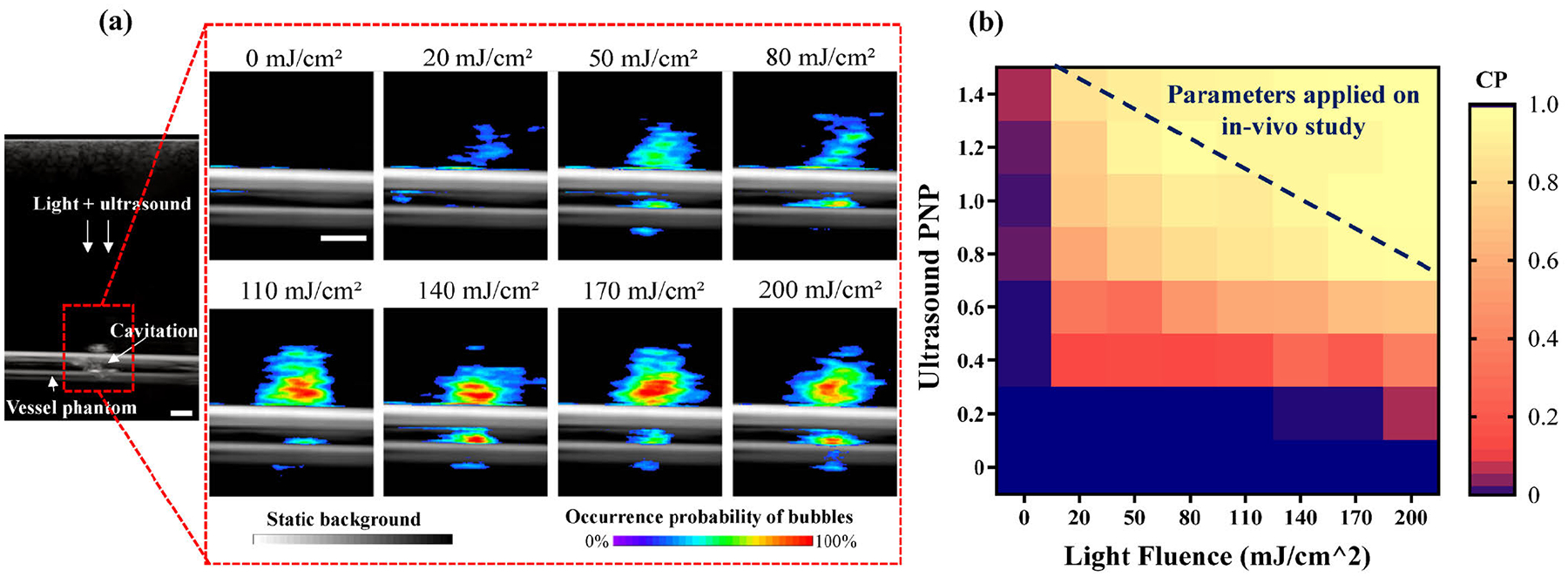
(a) Representative ultrasound images taken from the blood vessel treated with fixed ultrasound pressure at 0.6 MPa and varying light fluence from 0 to 200 mJ/cm^2^. White scale bar: 0.5 mm. (b) Cavitation probability (CP) quantified from ultrasound imaging of the blood vessel phantom.

The imaging result from the ultrasound imaging system was able to confirm the cavitation activities generated by the concurrently applied light pulses and ultrasound bursts during PUT. The representative results in Fig. 3.a were acquired when the ultrasound pressure was fixed at 0.6 MPa while the light fluence was swept from 0 to 200 mJ/cm^2^. 100 B-mode frames were recorded within a fixed region of interest. Cavitation was considered to occur in any imaged pixel after masking the tube structure once the pixel intensity exceeded three standard deviations above the baseline measured at 0 mJ/cm^2^. When the light fluence increased, the detected cavitation activities also increased. Fig. 3.b shows the measured cavitation probability from the blood vessel phantom treated by a combination of different levels of ultrasound pressure and light fluence. The quantified cavitation probability at each point in this map was calculated by counting the number of frames with detected cavitation bubbles from a total of 100 frames of ultrasound images. Unlike the simple demarcation in simulation results shown in Fig. 1.c.2, the cavitation probability shown in Fig. 3.b demonstrated the stochastic nature of cavitation events.

The points along the dashed blue line in Fig. 3.b have the same level of cavitation probability. For instance, the light fluence of 20 mJ/cm2 plus the ultrasound pressure of 1.4 MPa yields a similar cavitation probability as the light fluence of 200 mJ/cm^2^ plus the ultrasound pressure of 0.8 MPa. This experimental result from the phantom study indicates again that similar cavitation activities can be achieved with weaker light fluence in combination with stronger ultrasound pressure (the situation in deep skin) or stronger light fluence in combination with weaker ultrasound (the situation in superficial skin), which is consistent with the finding in Fig. 1.c.2 from the theoretical modeling.

### B. IN VIVO STUDY

The experiment setup employed for in vivo PUT treatment is shown in Figure 3. While the focused ultrasound system was the same as the phantom study, additional adjustments were applied to ensure treatment efficiency on chicken wattle. A 3D-printed holder designed to fit the focused transducer was utilized to hold gel and degassed water for ultrasound coupling from the transducer to the target tissue. A central hole in the gel was reserved for passing of the light beam. This design, which was different from that in our previous study [21], facilitated that the light beam and the ultrasound wave for PUT could be delivered from the same side. Two layers of ultrasound coupling materials, i.e., gel and degassed water, were used to reduce the pathlength of the light beam in water which has a relatively strong optical absorption at the wavelength of 1064 nm. Detailed dimensions and descriptions of the 3D-printed holder are included in the supplementary materials. To verify the acoustic coupling from the transducer to the target tissue, light was turned on and the transducer collected photoacoustic signals, which were then amplified by a pulser/receiver (PR 5072, Olympus, Japan) and shown on an oscilloscope. The amplitude of the photoacoustic signal was used as a control point for treatment at each site, with similar signal amplitude indicating similar acoustic coupling efficiency.

The light and ultrasound parameters used during PUT treatment of blood vessels in chicken wattle are listed in Table 1, which were initially selected based on our previous in vivo studies [21] and further adjusted according to the theoretical modeling and in vitro phantom studies for treated of deep vessels. Via a 6 mm diameter beam, the light pulse energy delivered to the top surface of the wattle was 80 mJ, leading to a light fluence of 283 mJ/cm2. After optical attenuation through the chicken wattle, the light pulse energy at the bottom of the wattle measured using a laser energy meter (843-R, Newport Corporation, Irvine, CA) was 16 mJ. With an estimated beam size of 6 mm in diameter, the light fluence at the bottom of the wattle was 56 mJ/cm2. To promote the treatment of deep vessels, the ultrasound wave was focused at the bottom of the wattle. Based on the precalibration in a water tank using a calibrated needle hydrophone (HNC-1500, Onda, Sunnyvale, CA), the ultrasound pressure arriving at the top surface of the wattle was 0.91 MPa, while the ultrasound pressure arriving at the bottom surface of the wattle was 1.36 MPa. The −6 dB focal width of the ultrasound beam was 6 mm.

Before each treatment, the laser was turned on and the resulting photoacoustic signal was received by the ultrasound transducer. This arrival time of the signal was used to determine the minimum delay needed between the laser and ultrasound triggers, serving as a reference for synchronization. More details about this approach have been reported in our previous publication [9]. We have also demonstrated, through both experimental and theoretic studies, that there is no significant change in cavitation threshold as long as the laser pulse is synchronized to the first half of the ultrasound rarefaction cycle [22], [23], [26]. To ensure that vessels at various depths receive optimal treatment, the synchronization between the light pulse and the ultrasound burst was swept across the entire 4-minute treatment session. This sweeping was achieved by adjusting the triggering delay by 500 ns every minute. For instance, the light pulse was synchronized to the negative peak of an ultrasound cycle at the skin surface during the first minute of treatment. During the second minute, the light pulse was delayed by an additional 500 ns. In this way, the light pulse was synchronized to the negative peak of an ultrasound cycle at the depth of 500 ns × 1.5 mm/*μ*s = 0.75 mm beneath the skin surface. This synchronization between the light pulse and the negative peak of an ultrasound cycle would shift to 2.25 mm depth during the 4th minute of the treatment session.

**FIGURE 4.**
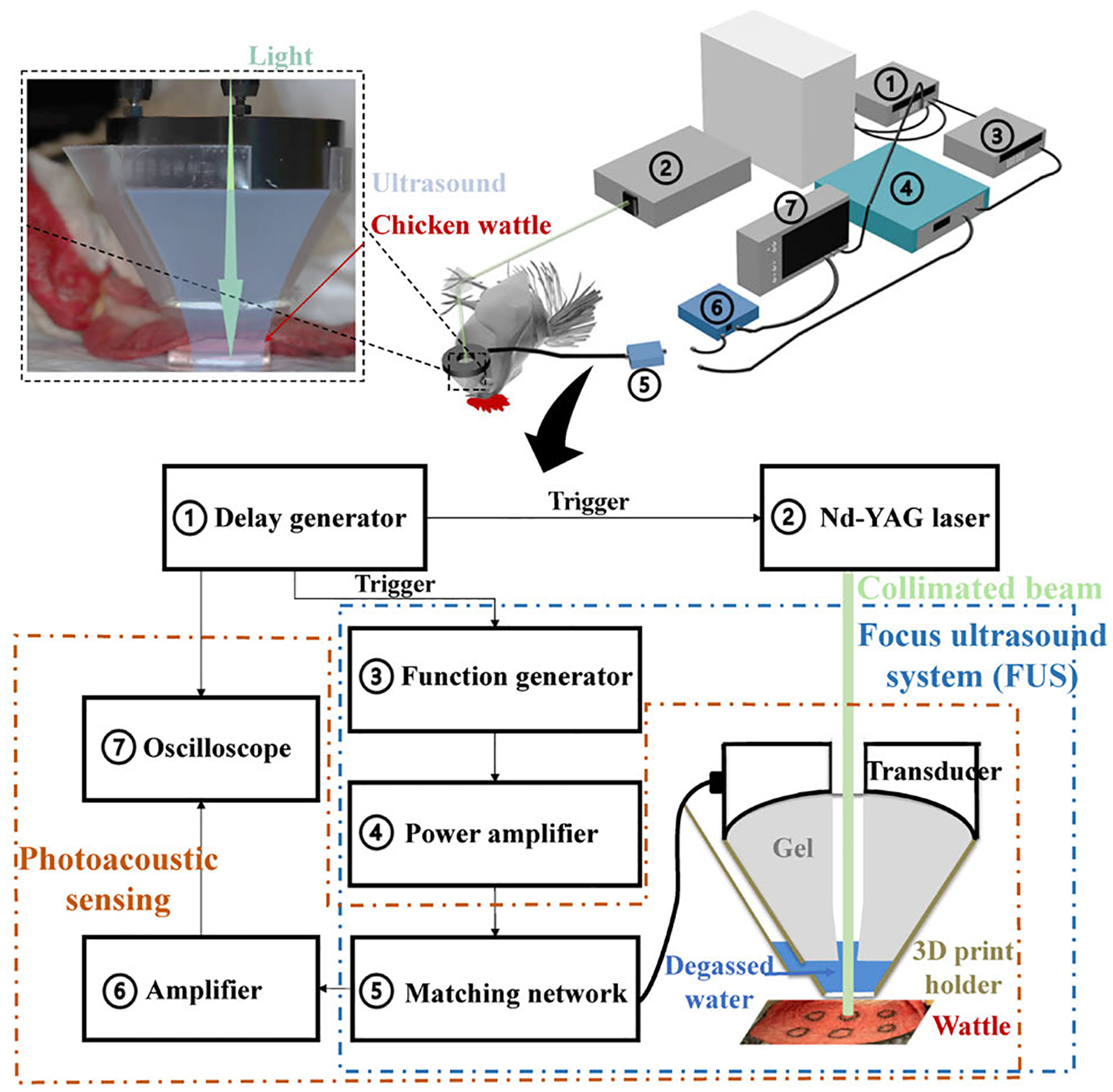
Setup of in vivo PUT treatment of the blood vessels in chicken wattle.

All the animal handling procedures were carried out in compliance with the protocol approved by the Institutional Animal Care and Use Committee (IACUC) at the University of Michigan (Protocol number: PRO00009939; PI: Xueding Wang). Using the parameters shown in Table 1 and the setup shown in Fig. 4, PUT treatment was conducted on four chicken wattles with three treatment spots on each wattle. Before and during the entire course of the treatment, chickens were anesthetized with an intramuscular injection of a cocktail of ketamine (15 mg/kg) and xylazine (0.6 mg/kg). The 3D-printed holder was sealed with a plastic membrane at the bottom, and ultrasound coupling gel was applied between the membrane and the chicken wattle to ensure good ultrasound delivery to the wattle.

To evaluate treatment outcomes, all treated areas on the chicken wattles were imaged using a polarized dermoscope (Canon EOS camera-based, Canfield Scientific) and a spectral domain optical coherence tomography (OCT) system (TEL 321 Telesto, Thorlabs). For comparison, an additional untreated area on each wattle was also imaged using the OCT system. OCT-angiography (OCT-A) images were generated using speckle variance analysis over three repeated B-scans per position. Images were taken at multiple time points, including before treatment (control), and at Day 1 and Day 7 after treatment. The imaging depth used for OCT-A analysis was 150–300 *μ*m, and all scans were acquired at 8 mm × 8 mm using a fixed color scale. Vessel density was estimated from total OCT-A signal intensity across the entire scan area. With the OCT-A images acquired at different time points and pre-processed by ThorImage^®^, quantitative analyses of vascular density and its change in response to the treatment were conducted by using the method described in a previous study [21].

To further investigate treatment efficacy (including depth) and safety, wattle tissues were harvested after the chickens were euthanized at Day 7 post-treatment. The tissues for histology and immunohistochemistry (IHC) were stained with hematoxylin and eosin (H&E), CD31 antibodies, and Russell-Movat Pentachrome (RMP) by the In-Vivo Animal Core in the Unit for Laboratory Animal Medicine at the University of Michigan. H&E stained slides were used to assess treatment depth by measuring the depth of inflammation and vascular necrosis performed by a pathologist. CD31 stained slides were used to quantify treatment efficacy by comparing the vascular densities inside and outside the treated area. RMP stains were utilized to assess the safety of the treatment by measuring the collagen densities inside and outside the treated area. The detailed staining protocols and the quantification methods are provided in the Supplementary Information 2.

## IV. RESULT

**FIGURE 5.**
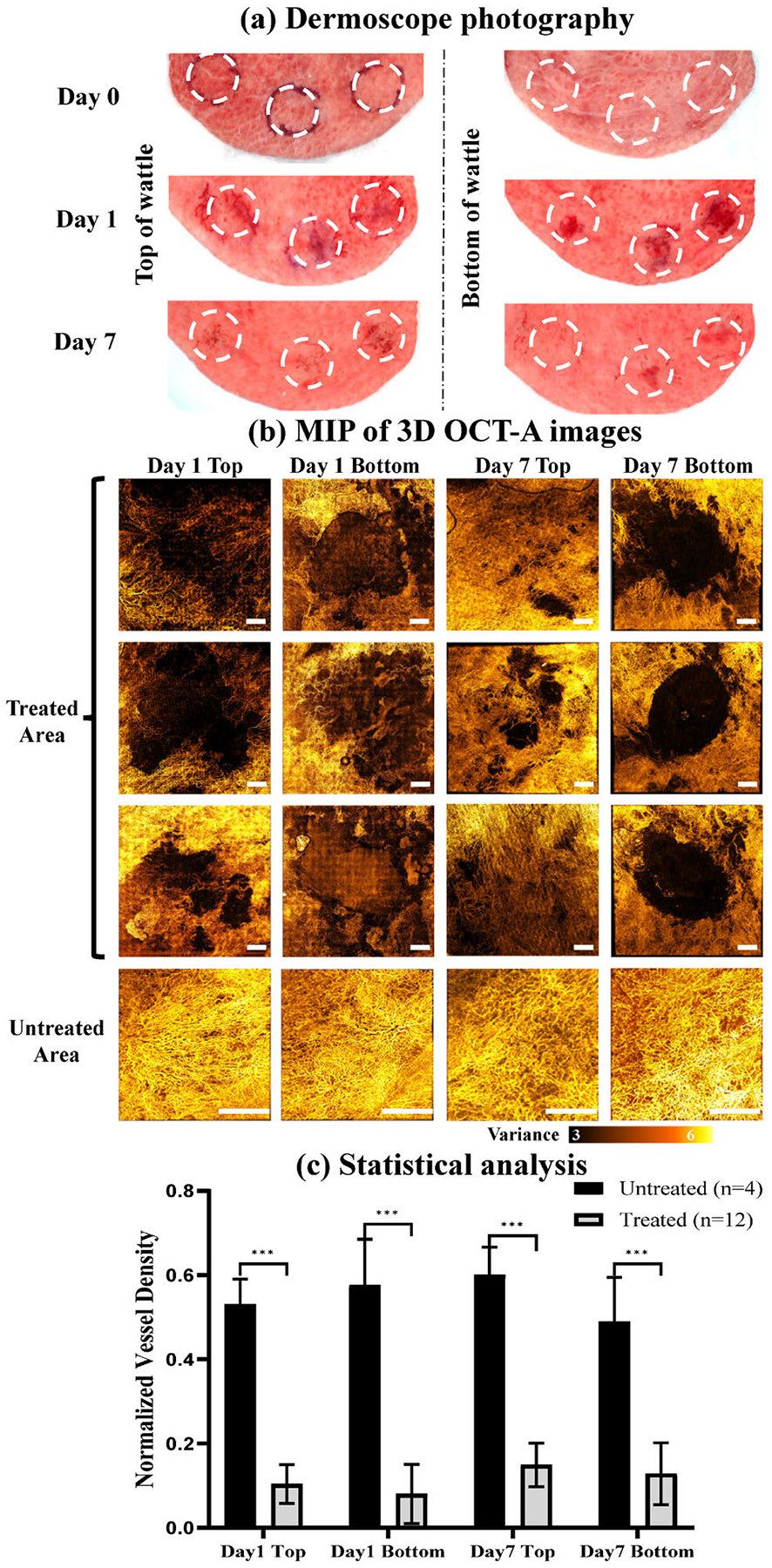
Representative treatment outcome from a chicken wattle treated with PUT. (a) Polarized dermoscope images of the chicken wattle with three PUT treatment areas (labelled by white dash circles) taken before and at different time points (Day 1 and Day 7) after the treatment. (b) Maximum intensity projection images of 3D OCT-A of the three treatment areas and one untreated area (control) on the same chicken wattle. White scale bar: 1 mm. (c) Normalized vessel density measured by OCT-A of the regions treated with PUT ( n = 12 ) and untreated regions ( n = 4 ) at Day 1 and Day 7 after treatment, represented by mean ± SD. ***P < 0.001 for paired t-test between the measurements from untreated regions vs. treated regions, indicating that the treatment reduced the vessel density at both the top and the bottom sides of the wattles.

The Fig. 5.a displays the photographs of wattle skin taken using a skin imaging camera immediately before treatment (Day 0), immediately post treatment (Day 1), and at Day 7 post-treatment. All the three regions marked by the white dashed circles received the same PUT treatment. A clearly visible change in color can be observed at Day 1 from both the top and the bottom sides of the wattle, suggesting that the treatment effect penetrated the entire chicken wattle.

To validate blood flow changes as the treatment outcomes from PUT, OCT-A images acquired from both the top and the bottom sides of the treated areas were compared with an untreated area as the control. As shown in Fig. 5.b, blood perfusion ceased immediately after PUT treatment in all the treated regions, and this cessation of blood perfusion persisted for the entire observation period of 7 days post-treatment. Quantitative assessment of PUT treatment outcomes was performed by analyzing the vessel density map generated from the 3D OCT-A images that were acquired before and at various time points post-treatment, as shown in Fig. 5.c. Each data point represents the mean ± standard deviation (SD) for each group. For the PUT treatment group, vessel density was significantly less than that in untreated regions at both the bottom and the top sides of the wattles at different time points post-treatment. At Day 7, the vessel densities at the top and the bottom sides of the wattles were reduced by 45.20% and 36.06%, respectively, when compared with the untreated regions, indicating good efficacy and depth of PUT in removing the vessels.

**FIGURE 6.**
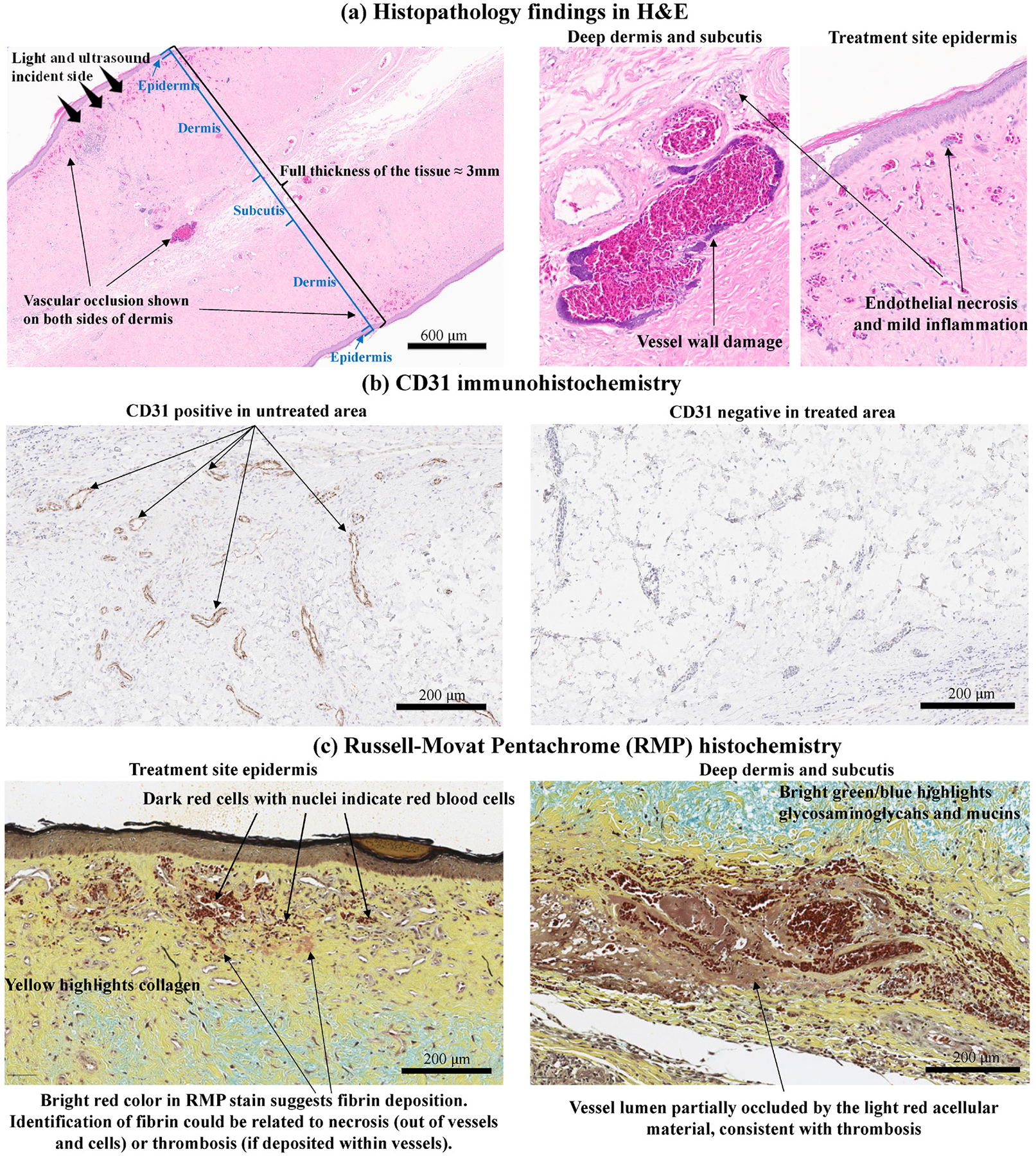
Representative H&E histology, CD31 immunohistochemistry, and RMP histochemistry photos from a chicken wattle harvested at Day 7 after PUT treatment.

**FIGURE 7.**
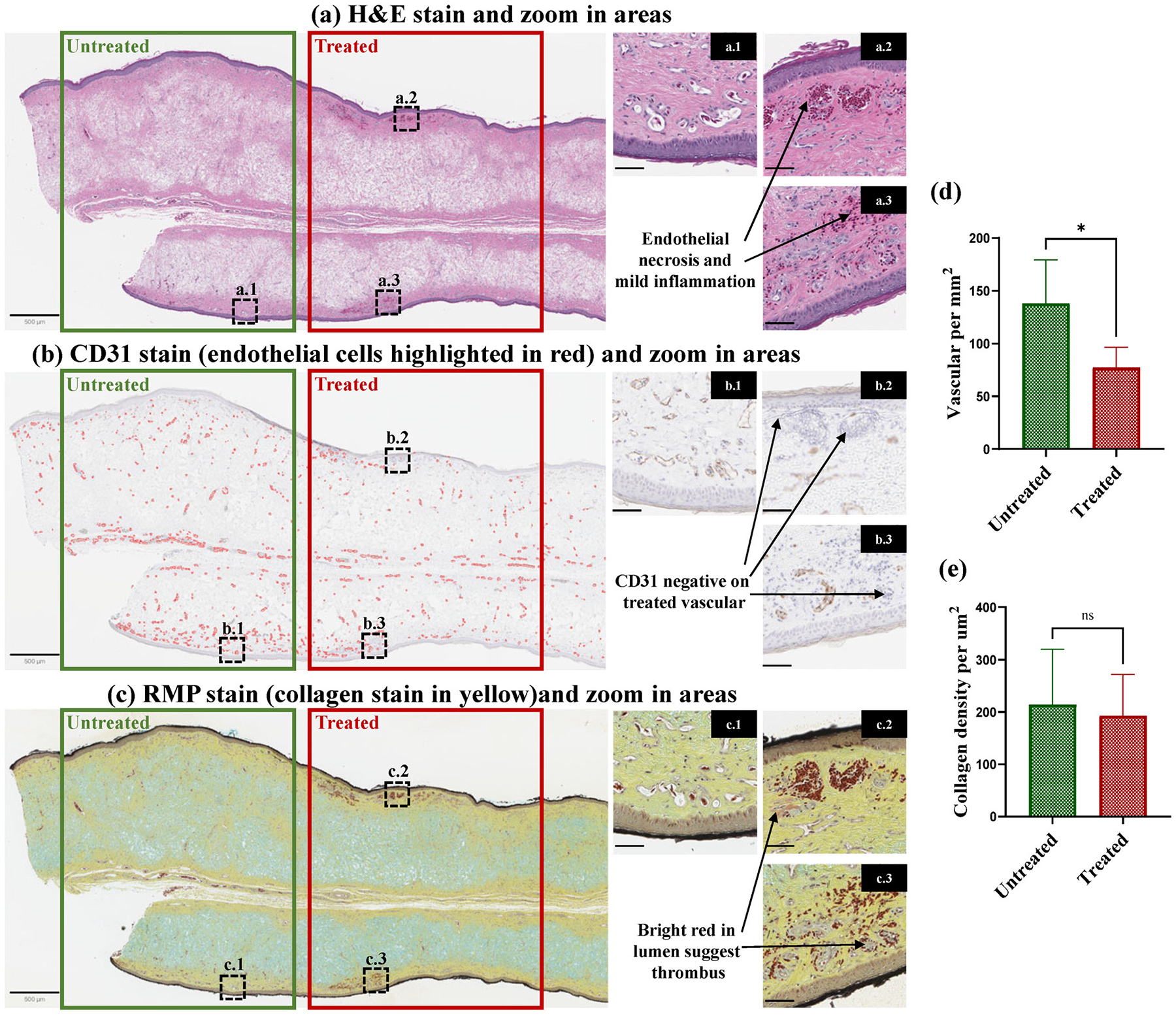
Histopathological analyses across the entire section of a chicken wattle conducted at Day 7 post-treatment. (a) H&E stained section, including the original photo and the zoom-in photos from two treated areas (a. 2 from the top and a. 3 from the bottom of the treated region) vs. an untreated area (a.1) as the control. Endothelial necrosis can be seen in both the top (a.2) and the bottom (a.3) of the treated region. (b) CD31 stained IHC from an adjacent section, including the original photo and the zoom-in photos from the same three areas (b. 2 and b. 3 from the top and the bottom of the treated region, and b. 1 from the untreated area). In the original photo, the endothelia cells are highlighted in red. In the zoom-in photos, original brightfield images demonstrate CD31 negative on treated vessels. (c) RMP histochemical stain from an adjacent section, including the original photo and the zoom-in photos from the same three areas (c. 2 and c. 3 from the top and the bottom of the treated region, and c. 1 from the untreated area). Thrombus in vessel lumens (bright red) in the treated areas can be seen, while no obvious change in collagen (yellow) is noticed. (d) Quantified vascular densities in the treated tissues vs. the untreated tissues by analyzing the IHC CD31 results. p < 0.05 when comparing the two groups via a paired t -test (n = 10 for each group). (e) Quantified collagen densities in the treated tissues vs. the untreated tissues by analyzing the RMP results. No statistically significant difference is noticed when comparing the two groups ( n = 6 for each group). For original photos, scale bar = 500*μ* m. For zoom-in photos, scale bar = 50*μ* m.

To further evaluate the efficacy and safety of PUT treatment, histopathological analyses were performed across the entire sections of chicken wattles at Day 7 post-treatment. Adjacent tissue slides were stained with H&E, IHC CD31, and histochemical RMP, as described in the Supplementary Information 2. Fig. 6 shows the representative photos of H&E, CD31, and RMP stained slides. The unique architecture of chicken wattle can be seen in Fig. 6.a as well as Fig. 7.a. The full thickness of the tissue comprises epidermis on both sides, with dermis underneath on both sides, and a very scant amount of subcutis in the center. As a result of PUT treatment, acute vascular necrosis and mild inflammation were noted in multiple capillary layers in the superficial dermis at both sides, leading to vascular occlusion. PUT treatment depth in the chicken wattle, which has a very dense vasculature, is up to 3 mm, given the treatment effect observed across the full thickness of the harvested chicken wattles (n=12, thickness of mean ± SD 2.51 ± 0.21 mm).

Fig. 6.b demonstrates a closer look of the CD31-positive endothelial cells formed blood vessel wall found in the untreated area, while CD31-negative area could be found in the PUT treated area. Additionally, as shown in Fig. 7.b, on the same slide, CD31-positive endothelial cells with clear vessel lumens observed in the untreated tissues, whereas endothelial cells in the treated area were CD31-negative on both sides of the chicken wattle, indicating the vascular damage across the entire wattle induced by PUT treatment. The RMP stained collagen in yellow and thrombus in pink are illustrated in Fig. 6.c. Fig. 6.c along with Fig. 6.c shows that, in the RMP stained sections, the collagen within the treated area retained a structure and morphology similar to those in the untreated area, indicating no collateral collagen damage induced by PUT treatment. Bright red areas were found in vessel lumens in both the top and the bottom layers of the wattle, suggesting thrombus formation after treatment. The CD31 results and the RMP results were further utilized to quantify the vascular densities and the collagen densities, respectively, in both the untreated and the treated tissues. The quantified vascular densities in the treated vs. the untreated wattle tissues are shown in Fig. 7.d, which indicates that a single session of PUT treatment led to an average reduction of 43.5% in vascular density. p<0.05 was achieved when comparing the treated and the untreated tissues via a paired t-test (n=10 for each group). The quantified collagen densities in the treated vs. the untreated wattle tissues are shown in Fig. 7.e. No statistically significant difference was noticed when comparing the treated and the untreated tissues via a paired t-test (n=6 for each group), confirming that there was no collateral collagen damage induced by PUT treatment.

## V. DISCUSSION

The treatment outcomes of PWS have not been improved over the past three decades [14]. The primary reason for many suboptimal therapeutic outcomes is believed to be the insufficient treatment depth associated with the PDL as the current standard clinical method. In this study, we demonstrated that PUT, with a unique capability to treat deep cutaneous vessels, could have significant advantages over traditional PDL therapy. The enhanced treatment depth is attributable to the synergistic use of nanosecond light pulses and ultrasound bursts to induce localized cavitation to damage blood vessels. While laser light fluence in deep tissue is low, ultrasound energy can penetrate deep tissue effectively and deliver sufficient ultrasound pressure to disrupt the vasculature. Via a study in a clinically relevant chicken wattle model, the treatment efficacy and safety of PUT in removing cutaneous vessels were validated by both OCT-A imaging and standard histopathological evaluations. It was demonstrated that PUT, by combining safe light fluence and safe ultrasound pressure, can eliminate the blood vessels with a depth up to 3 mm without causing collateral thermal damage in surrounding tissues. The main goal of the current study is to show that PUT can achieve deep treatment. We did not present *in vivo* data for laser-only and ultrasound-only control groups in the current study, which, however, has been reported in our prior publication [21] with the same parameter ranges. The previous results demonstrated no significant treatment effects from the laser-only and ultrasound-only control groups, supporting the importance of the synergistic mechanism of PUT.

The ultrasound imaging system used in the current study is a clinical system and cannot be synchronized with our laser system. Due to this limitation, we continuously acquired 100-B-mode frames to cover the entire sonication period of FUS in the cavitation probability study. We acknowledge that this approach could overestimate cavitation probability in high probability regions because bubbles may persist across consecutive frames. This effect may be mitigated by the flowing blood in the tube, which may carry away the formed bubbles. Further study will be needed to achieve a more precise quantification.

The unique mechanism of PUT provides flexibility and potential for adjusting the treatment parameters to achieve optimal outcomes in different cases. Since both light and ultrasound contribute to the treatment outcome, similar treatment effects may be achieved in various parameter combinations. Theoretical modeling and *in vitro* experiments validated that cavitation could be successfully induced under different combinations of light fluence and ultrasound pressure, e.g., weaker light fluence plus stronger ultrasound pressure, or stronger light fluence plus weaker ultrasound pressure. In addition to the flexibility in selecting light fluence and ultrasound pressure, factors such as laser wavelength, ultrasound frequency, and their synchronization also play critical roles in treatment outcomes. This study employed a sweeping synchronization strategy to ensure that optimal synchronization covered various tissue depths throughout the procedure. In the future, with the lesion depth and range measured through OCT-A [27], [28] or photoacoustic imaging [29], PUT’s synchronization strategy could be refined by fixing synchronization to target the lesion depth accurately, thereby maximizing treatment potential.

It is worth noting that the treatment depth of PUT may not be limited to 3 mm. The 3 mm treatment depth demonstrated in this study was limited by the chicken wattle model which has a maximal thickness of about 3 mm. Therefore, it is highly likely that PUT can be demonstrated to have a treatment depth of more than 3 mm in future clinical studies, especially after treatment parameters are further optimized. A limitation of this study, as demonstrated in the histopathological evaluations, is that the deep treatment regions showed uneven distribution of therapeutic effects. The observed variability, particularly at the bottom of the chicken wattle, is primarily attributed to anatomical differences, such as local variations in tissue thickness. Thicker regions resulted in increased optical attenuation and altered synchronization between laser and ultrasound, leading to less consistent cavitation. This problem may be addressed by further adjusting the treatment parameters, sweeping the treatment region determined by the overlapping light and ultrasound beams over the skin surface, or using image-guided treatment. Another limitation of this study is that the therapeutic effects were only studied for a period of one week. Despite the fact that vessel perfusion in the chicken wattle was stopped immediately post-treatment and persisted 7 days after treatment, it is possible that reperfusion could occur in treated regions with longer follow-up. Similar recurrence of PWS is often seen after PDL treatment. It has been hypothesized that PWS recurrence may be associated with the induction of angiogenesis and the revascularization of lesions [30], [31]. Because of PWS recurrence, current PDL therapy for PWS usually requires multiple treatment sessions, with standard intervals ranging from 6 to 12 weeks [32], [33].

Both OCT-A and dermoscopy were used to evaluate the *in vivo* treatment outcomes in the current study. Please note that the OCT-A images capture microvascular flow, whereas dermoscopy highlights surface-level changes such as discoloration and textural differences. As a result, discrepancies in evaluation might be possible when comparing the results from OCT-A and dermoscopy. For instance, the OCT-A images in Fig. 5 suggest more extensive damage at the bottom of the wattle at Day 7, which seems different from the dermoscopy results. While the top region of the wattle received more direct laser energy exposure, the bottom region received more ultrasound energy by our design and may have exhibited delayed vascular remodeling, inflammation, or tissue remodeling processes, which is a possible reason that OCT-A images at Day 7 show more extensive damages at the bottom of the wattle.

It is also important to recognize that clinical translation will require further parameter refinement based on skin tone. Melanin content varies significantly across the Fitzpatrick scale, influencing light absorption and, consequently, effective treatment depth. Darker skin types may require lower light fluence or modified ultrasound pressure to avoid overtreatment or reduce variability in cavitation induction. In addition, a side-by-side comparison of the treatment outcome from PUT with conventional PDL will be necessary in future studies to fully understand the potential benefits that can be brought by this new treatment method.

## VI. CONCLUSION

Via a study in a clinically relevant chicken wattle model of PWS in vivo, we demonstrated that PUT, by using spatiotemporally synchronized light pulses and ultrasound bursts, can effectively eliminate blood vessels with a treatment depth up to 3 mm without causing collateral thermal damage to the surrounding tissues. The light fluence utilized in this study was 10–100 times lower than that employed in traditional PDL therapy. A single PUT treatment session can induce 43.5% reduction in blood vessel density in the chicken wattle model of PWS.

## Supplementary Material

supp1-3604391

supp2-3604391

This article has supplementary downloadable material available at https://doi.org/10.1109/OJUFFC.2025.3604391, provided by the authors.

## Figures and Tables

**TABLE 1.** Put parameters for treatment of blood vessels in chicken wattle in vivo.

	Light			Ultrasound				
	Pulse Energy	Beam Diameter	Light Fluence	Peak Negative Pressure	Duty Cycle	Focal Width	Pulse Repetition Rate	Treatment Duration
At Top	80 mJ	6 mm	283 mJ/cm^2^	0.91 MPa	2 % (500 cycles)	6 mm	10 Hz	4 min
At Bottom	26 mJ	N/A	56 mJ/cm^2^	1.36 MPa				
