# Supplementary material for: Photo-Mediated Ultrasound Therapy (PUT) for the Treatment of Deep Cutaneous Vasculature
Source: IEEE Open J Ultrason Ferroelectr Freq Control. Author manuscript; Available in PMC 2025 Sep 20. (PMC12448061; doi:10.1109/ojuffc.2025.3604391)
Supplement: supp1-3604391 [file NIHMS2110738-supplement-supp1-3604391.docx]

**Supplementary Information 2 – Hematoxylin and eosin (H&E) stain, CD31 stain, histochemistry of Russell Movat pentachrome stain, and data analysis procedures**

Twelve samples of chicken wattle tissues were submitted to the Pathology Core of the Unit for Laboratory Animal Medicine (ULAM) at the University of Michigan. Each piece of tissue measured approximately 1 cm × 1 cm × 0.2 cm, with a diameter of 1.5-2 mm indentation at the center (treatment site). Gross pictures of these specimens were shared by the lab. All tissues were fixed in 10% neutral buffered formalin at a ratio of 10:1 fixative:tissue for more than 24 hours. Tissues were trimmed on the midline of the indentation site and processed to paraffin in an automated tissue processor (TissueTek VIP5, Sakura). Both halves of the sections were embedded in the same paraffin block. Tissues were sectioned on a rotary microtome at 4 µm thickness, with 3 sections (representing both halves) of the tissue per slide and 100 µm intervals between each section for a total of 4 slides. One of these slides per specimen was stained with hematoxylin and eosin (H&E) by standard methods on an automated histostainer (Autostainer ST5010 XL, Leica Biosystems). One of these was used for immunohistochemistry of CD31 stain. The remaining slide was used for histochemistry of Russell Movat pentachrome stain.

Full thickness of the tissue of chicken wattle is composed of epidermis on both sides, with dermis underneath also on both sides, and a scant amount of subcutis in the center. The depth of the lesions was measured from the surface of the epidermal necrosis, crossing dermis and subcutis, to the bottom of the lesion which might be at dermis on the other side of the tissue. The ratio was calculated as the depth of the pathology divided by the full thickness of the tissue in micrometer (µm).

Light microscopic evaluation was performed at magnifications ranging from ×20 to ×600. The evaluation was performed initially by a board-certified veterinary pathologist with peer review by a second board-certified veterinary pathologist. Representative images were taken from slides digitized on a digital slide scanner (Leica Aperio AT2 digital slide scanner, Leica Biosystems) at resolution of up to 0.5 µm/pixel (equivalent to 20× microscope objective).

Manual scoring was applied to evaluate the severity of necrosis and hyperplasia of the epidermis, necrosis and inflammation of the dermis, and vascular necrosis and inflammation of the subcutis. Depth of the necrosis was measured on digital slides viewed in ImageScope viewer.

**Immunohistochemistry of CD31**

The primary antibody used were the rat monoclonal anti-mouse endothelial cell marker CD31 (PECAM-1) (Cat No. DIA-310 (100µg), Clone SZ31, Dianova) at an optimized dilution of 1:50. Unstained sections were cut on a rotary microtome at 4 µm thickness and mounted on glass slides. Heat-induced epitope retrieval was performed in a pH6.2 buffer (DV2004, DIVA Decloaker, Biocare Medical) in a laboratory pressure cooker (DC2002, Decloaking ChamberTM, Biocare Medical) at temperatures alternating between 125°C (40 seconds) and 95°C (10 seconds) for a total of 40 minutes, then cooled for 15 minutes. Immunohistochemical staining was performed on an automated immunostainer (Biocare Intellipath, Biocare Medical). The protocol consisted of endogenous peroxidase quenching Peroxidazed 1, PX968, Biocare Medical) for 5 minutes and blocking of non-specific sites (Biocare Rodent Block M, RBM961, Biocare Medical) for 30 minutes. The primary antibody was applied at room temperature at the dilution specified above for 1 hour. Detection was performed using a biotin-free polymer-based detection system with rat probe (RTP629, Biocare Medica) followed by a Rat-on-Mouse Polymer-HRP (RTH630, Biocare Medical). Diaminobenzidine chromogen (DAB chromogen, IPK5010G80, BiocareMedical) was reacted for 5 minutes, followed by a DAB enhancer (IP sparkle, DS830G, Biocare Medical) for 1 minute. Slides were counterstained with hematoxylin (Biocare Medical), dehydrated through graded ethanols, cleared in xylene, and coverslipped using a permanent mounting media. One mouse tissue section containing intestine, uterus, and a lymph node, was applied as a positive control slide. One section of chicken wattle tissue was used for negative control, with Rat Negative Control Sera (Cat No. NC915, Innovex) applied as the primary antibody. Control and experimental slides were evaluated by a board-certified research veterinary pathologist with respect to staining compartment, cell-specificity, intensity, and presence/absence of background or artifact.

**Histochemical stain- Russell-Movat Pentachrome (RMP)**

Slides were deparaffinized, hydrated, and stained in elastic stain for 20 minutes. Differentiation was performed in Ferric Chloride, 2% for 15-20 dips, with progress monitored microscopically. Slides were then placed in sodium thiosulfate, 5% for 1 minute to remove excess iodine, and acetic acid, 1% for 2 minutes to equilibrate tissue. They were then stained with Alcian blue, 1%, pH 2.5 for 25 minutes, and Biebrich Scarlet-acid Fuchsin for 2 minutes. Differentiation was performed in phosphotungstic acid, 5% for 3 minutes, twice, with progress monitored microscopically. Slides were then placed in acetic acid, 1% for 1 minute, and Yellow Stain Solution for 15 minutes. Finally, slides were dehydrated, cleared in xylene, and mounted with Micromount.

**Digital slide assessment and quantitation**

Slides were digitized on a Leica Aperio AT2 digital slide scanner (Leica Biosystems) at a resolution of 0.25 µm/pixel (40x objective). Quantitative assessment of immunohistochemical staining was performed using the open-source program QuPath v0.5.1 ([github.com/qupath/qupath](https://github.com/qupath/qupath)). Detection parameters were optimized using the positive and negative control slides. Images were first annotated manually. Then, the annotated area was subjected to a pixel thresholder, which was either trained via Artificial Neural Network or by default, to measure the area of the positive objects, as well as to count the positive objects. Microsoft Excel and Graphpad Prism 10 were then used for data organization, visualization, and statistical analysis.
