# Supplementary material for: Photo-Mediated Ultrasound Therapy (PUT) for the Treatment of Deep Cutaneous Vasculature
Source: IEEE Open J Ultrason Ferroelectr Freq Control. Author manuscript; Available in PMC 2025 Sep 20. (PMC12448061; doi:10.1109/ojuffc.2025.3604391)
Supplement: supp2-3604391 [file NIHMS2110738-supplement-supp2-3604391.docx]

**Supplementary Information 1 – Theoretical modeling equations and parameters**

**Mechanism of PUT**

The underlying mechanism for PUT is cavitation induced by synchronized light pulses and ultrasound bursts. Previous theoretical studies on cavitation bubble dynamics and cavitation-induced stresses during PUT have identified three distinct phases during the interaction of light pulses and ultrasound bursts within blood vessels. The three distinct phases illustrated in Supplementary Figure 1 could be outlined as: 1) in the initial phase, a pulsed light induced photoacoustic wave converging at the center of the cylindrical shaped optical absorber, synchronized with the peak negative pressure of a ultrasound wave, triggers bubble nucleation; 2) in the subsequent phase, the formed bubble expands through rectified diffusion process in the ultrasound field; and 3) in the third phase, the bubble either breaks up which undergoes inertial cavitation or reaches a stable equilibrium radius and undergoes non-inertial oscillation. In the present study, we used an established theoretical model to investigate how light fluence and ultrasound peak negative pressure influence pre-existing bubbles during PUT by implementing the light and ultrasound parameters utilized in our current experiments.


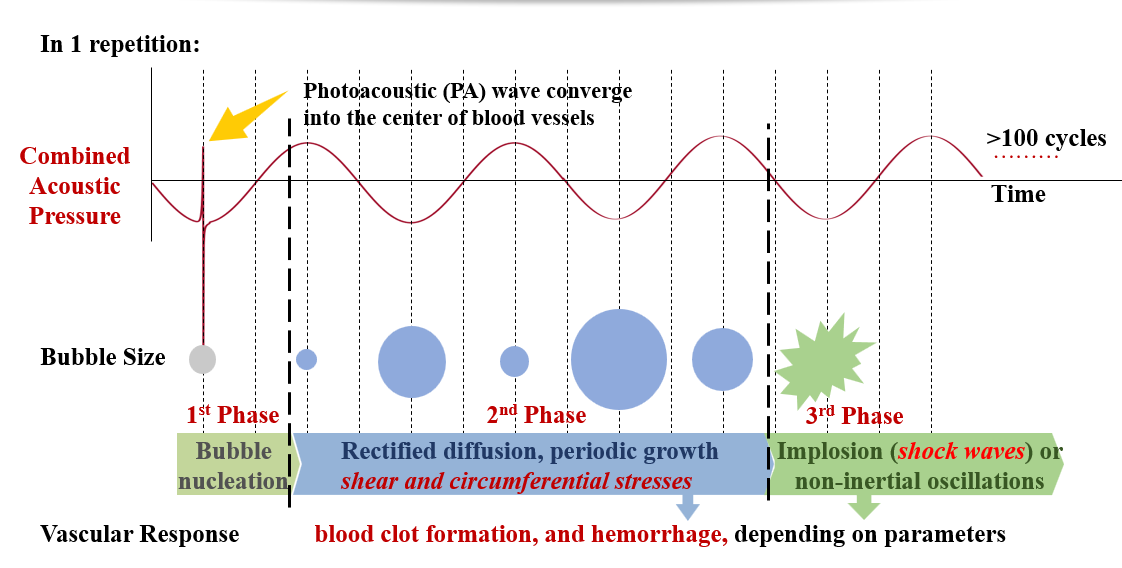


Supplementary Figure 1. An illustration of the hypothesis for the underlying treatment mechanism in PUT.

**Bubble Dynamic**

The single bubble dynamic mathematical model used theoretical model based on the Keller–Miksis, which has the form in **equation (1)**.

$$\left( 1-\frac{\dot{R}}{c} \right)R\ddot{R}+\frac{3}{2}\left( 1-\frac{\dot{R}}{3c} \right)\dot{R}^{2}=\frac{R}{\rho c}\frac{d}{dt}\left[ p_{B} \right]+\frac{1}{\rho}\left( 1+\frac{\dot{R}}{c} \right)\left( p_{B}-p_{\infty}-p\left( t+\frac{R}{c} \right) \right) (1)$$

In this equation, R is the bubble radius, dots denote time derivatives, t is time, c is the sound speed in the surrounding medium, ­ $\rho$ is the density of the surrounding medium, *p_∞_* is the pressure at infinity, and $p_{B}$ is the pressure at the surrounding medium side of the interface between the medium and the bubble.

$$p_{B}=p_{g}-\frac{2\sigma}{R}-\frac{4\mu}{R}\dot{R} (2)$$

**Equation (2)** gives the formula of *p_B_*, where *σ* is the surface tension coefficient, *μ* is the viscosity of the fluid, and *p_g_* is the pressure inside the bubble.

$$n=n_{0}-4(\pi D)^{\frac{1}{2}}\int_{0}^{\tau} F\left( \tau^{'} \right)\left( \tau-\tau^{'} \right)^{-\frac{1}{2}}d\tau^{'} (3)$$

To consider the rectified diffusion, *p_g_* can be calculated from Eller and Flynn’s zero-order solution to the diffusion equation, expressed as **equation (3)**, where *D* is the diffusion constant of the gas in the liquid, *n_0_* is the number of moles of gas initially present in the bubble. *τ* and $F\left( \tau\right)$ are the new time variable and function defined using following **equations (4) &(5)**.

$$\tau=\int_{0}^{t} R^{4}\left( t^{'} \right)dt^{'} (4)$$

$F\left( \tau\right)=C_{0}\left( \frac{p_{g}}{p_{\infty}} \right)-C_{i}$ (5)

In this formulation, *R* is the radius oof bubble, *C*_0_ is the saturation concentration of the gas in the liquid, *C_i_* is the initial concentration of gas in the liquid far from the bubble. Due to *p_g_* is a function of *n*, **equations (3)** need to be coupled with following equations and solved simultaneously.

$$p_{g}=\left( p_{\infty}+\frac{2\sigma}{R_{0}} \right)\left( \frac{n}{n_{0}} \right)\left( \frac{R_{0}}{R} \right)^{3\eta}\left( \frac{R_{0n}}{R_{0}} \right)^{3\left( \eta-1 \right)} (6)$$

$$p_{\infty}+\frac{2\sigma}{R_{0n}}=\frac{3nR_{g}T}{4\pi R_{0n}^{3}} (7)$$

In **equation (6)**, *η* is the polytropic exponent of the gas, *R*_0_ is the initial equilibrium radius of the bubble, and *R*_0_*_n_* is the time-varying equilibrium bubble radius calculated using ideal gas law expressed in **equation (7)**, where *R_g_* is the universal gas constant and *T_a_* is the absolute temperature.

**Photoacoustic Pressure in Cylindrical Optic Absorber**

In **equation (1)**, the term $p\left( t+\frac{R}{c} \right)$ is the applying pressure, which is composed of the applied ultrasound pressure and the photoacoustic (PA) pressure produced by a light pulse.

$$p\left( \overset{-}{r},t \right)=\frac{\int_{-\infty}^{+\infty} e^{-\left( 2t^{'}/T \right)^{2}}p_{\delta}\left( \overset{-}{r},t-t^{'} \right)dt^{'}}{\int_{-\infty}^{+\infty} e^{-\left( 2t^{'}/T \right)^{2}}dt^{'}} (8)$$

$$p_{\delta}\left( \overset{-}{r},t \right)=\frac{1}{4\pi c^{2}}\frac{\partial}{\partial t}\left[ \frac{1}{ct}\int d{\overset{-}{r}}^{'}p_{0}\left( {\overset{-}{r}}^{'} \right)\delta\left( t-\frac{\left| \overset{-}{r}-{\overset{-}{r}}^{'} \right|}{c} \right) \right] (9)$$

$$p_{0}\left( r \right)=\Gamma\mu_{a}F\left( r \right) (10)$$

$$F\left( r \right)= \mu_{a}F_{0}e^{-\mu_{a}l} (11)$$

As we consider the light pulse is a Gaussian temporal profile, the distribution of initial pressure produced by the light pulse can be calculated by **equation (8),** where *T* is the light pulse width defined at the full width of half maximum, and *p_δ_* is the initial pressure distribution of the absorbing object. Considering the attenuation of light radiant exposure across a blood vessel, the pressure response for a delta heating of an arbitrary absorbing object is given by **equation (9)**, where *p_o_* could be calculated using **equation (10)** as the initial photoacoustic pressure distribution. The initial photoacoustic pressure across the profile of blood vessel can be acquired after considering attenuation using **equation (11)**. $\Gamma$ is the Gruneisen constant, $\mu_{a}$ is the optical absorption coefficient, $F_{0}$ is the initial laser fluence at surface and $F\left( r \right)$ is the local laser fluence inside blood vessel after $l$ travel distance from the surface.

**Representative Simulation Results**

The representative simulation results shown in the manuscript demonstrate theoretical photoacoustic pressure and bubble dynamics in a blood vessel from the model. We assume an air bubble in blood with given a initial equilibrium radius of the cavitation nuclei R_0_ (50-200 nm). In all simulations, values for the constants are: *Γ* = 0.2, *μ_a_* = 10 cm^−1^, *c* = 1500 m·s^−1^, *ρ* = 1000 kg·m^−3^,  *p_∞_*­ = 1.01 × 10^5^ Pa, ­ *σ* = 0.0725 N·m^−1^, *μ* = 0.005 Pa·s, *D* = 2 × 10^−9^ m^2^ s^−1^, *η* = 1.4, *C_i_/C_0_* = 1, *R_g_* = 8.3145 J (mol · K)^−1^, and *T* = 293.15 K.
